# Dislocation of Hip

**Published:** 1828-12

**Authors:** 


### DISLOCATION OF HIP.

### *Dislocation of the right Hip, with compound Fracture of the left Leg.*

William Wanklin, æt. twenty-three, was received into the Worcester Infirmary, February 27th, under the care of Mr. Sheppard, in consequence of a severe injury he received by a quantity of gravel falling on him while working in a pit. The accident occurred in the neighbourhood of Malvern, a distance of eight miles, and he was sent to the hospital in an open cart. The day was very wet and cold, and the patient, on his arrival, was faint and much exhausted. On examination, the right hip was found to be dislocated, and there was also a compound fracture of the left leg. The present state of the patient being judged most favorable to the reduction of the hip, the fractured limb was temporarily secured in splints, the patient placed on a table, and the dislocation (which was in the ischiatic notch) was reduced by extension with the pulleys in less than ten minutes. The patient was now removed to bed, the bones of the leg placed in apposition, and the wound, being small, and not much contusion surrounding it, united by the first intention. The patient was discharged cured in six weeks after the accident.

